# The Struggle for Technology: Towards a Realistic Political Theory of Technology

**DOI:** 10.1007/s10699-015-9470-7

**Published:** 2015-10-29

**Authors:** Peter-Paul Verbeek

**Affiliations:** Department of Philosophy, University of Twente, Enschede, Netherlands

**Keywords:** Neo-Marxism, Political philosophy of technology, Postphenomenology, Empirical philosophy

## Abstract

Pieter Lemmens’ neo-Marxist approach to technology urges us to rethink how to do political philosophy of technology. First, Lemmens’ high level of abstraction raises the question of how empirically informed a political theory of technology needs to be. Second, his dialectical focus on a “struggle” between humans and technologies reveals the limits of neo-Marxism. Political philosophy of technology needs to return “to the things themselves”. The political significance of technologies cannot be reduced to its origins in systems of production or social organization, but requires study at the micro-level, where technologies help to shape engagement, interaction, power, and social awareness.

## Introduction

How to do political theory of technology? This is the main question that arises from Pieter Lemmens’ erudite and at the same time rather gloomy political analysis of information technology. At a high level of abstraction, Lemmens offers us a tour along several neo-Marxist approaches to technology and our economic system, in order to conclude that we urgently need to regain our ‘social autonomy’. The question remains, though, if this analysis lays bare all dimensions of the political significance of information technologies.

Digital technologies, according to Lemmens—or at least according to the authors he cites; the difference is not always clear—create a new proletariat, which can be called a ‘cognitariat’. They control both production processes and consumption processes, and rather than liberating people they enslave them. The central effect of this ‘machinic enslavement’ is that digital technologies do not only affect our individual lives by creating distraction and mass consumption, but also our ‘social autonomy’: our capacity to act as a society. And in doing so, they weaken our capacities of resistance. The only answer to this is to “adapt” technologies “to what we collectively decide to be a life worth living” and to “adopt” technologies “as weapons for reconquering our collective autonomy in the light of an increasingly totalitarian capitalism”.

Lemmens’ approach raises two fundamental questions as to how to do political philosophy of technology. First of all, the question arises to what extent a political theory of technology needs to base itself on empirical research of technology. For that reason, I will discuss Lemmens’ approach in relation to the so-called ‘empirical turn’ in philosophy of technology. Second, the question arises what the limitations of a neo-Marxist perspective on technology are. Alternative frameworks reveal other forms of political significance of information technologies, and result in alternative conclusions.

## Political Philosophy and the Empirical Turn

Lemmens’ analysis of information technology seems to leave us with two alternatives: enslavement versus reconquering autonomy. “Digital technologies” affect our self-consciousness and our social consciousness, which undermines our collective agency and social autonomy, and which asks for subversive action to turn the tide. In a certain way, this approach resembles the classical analyses in philosophy of technology, which also tended to focus on the potentially alienating role of technology, and which conceptualized ‘Technology’ as a broad social and cultural phenomenon, rather than investigating actual technologies. Along comparably abstract and gloomy lines, Lemmens makes firm claims about “fixed capital residing in the nervous systems of living labor”, “the increasing integration of the human mind into the digital circuits of capital”, and the “discrepancy between cyberspace” and “cybertime”, which “induces all kinds of psychopathologies” and “erodes the subject’s sensibility and affectivity due to the constant pressure to adapt one’s psychic apparatus to the codes and rhythms of the network.” Technology is taken as a broad category, and its impact is seen as essentially negative.

The ‘empirical turn’ in philosophy of technology (Achterhuis 2001) aimed to provide a more nuanced alternative to the overly abstract and generalizing character of classical approaches. Philosophical abstraction, it holds, should be linked to the study of actual technologies and technological developments, in order to ensure an engaged, well-informed and relevant analysis. Rather than speaking about technologies as manifestations of some underlying principle, philosophy of technology should investigate the implications of concrete technologies for philosophical themes like knowledge, morality, and politics.

Lemmens’ approach is deep and rich, but it is certainly not empirical. He interprets the work of other authors, most notably the work of Bernard Stiegler, and integrates these approaches into a political theory of technology. Rather than constructing a philosophical framework in close relation to actual information technologies, he builds upon psychoanalytic and Marxist frameworks that were developed way before information technologies came into being. To be sure, this does not make these frameworks useless. But such an analysis at least requires an explicit problematization of the very possibility to speak in these terms about information technologies.

The ‘empirical turn’ has brought a sensitivity for philosophical issues ‘from within’ rather than ‘from outside’ technologies. To what extent do concepts like “organic nervous systems of the cognitariat” and “libidinal energies” help to clarify the role of actual information technologies in society? And could there also be other impacts than the “de-autonomization” that is so central in the neo-Marxist frameworks discussed? Reversing the perspective, by starting from actual technologies rather than pre-given philosophical frameworks, may reveal a much more nuanced picture. Even though there certainly is a growing dependence on large companies like Microsoft, Apple and Google, and on their products, which have a profound influence on our daily lives, these very technologies can also be the source of new forms of social agency and self-awareness.

It cannot be denied that new information technologies challenge our attention, our concentration, our independence, and our creativity. But at the same time there is a growing awareness of these impacts, which makes people increasingly deal with them in a critical and creative way. Moreover, these new information technologies are themselves also the source of new forms of political awareness, as the so-called “Facebook revolutions” in Iran, Egypt, and Tunisia have shown, as well as the Tahrir square protests in Turkey. Instead of a libidinal inferno of consumption and distraction, information technologies played a much more productive role in these situations. Even though there are good reasons not to overestimate the actual foundational role of social media in these protests (cf. Morozov 2011), it cannot be denied that information technologies also open up new political spaces, rather than merely closing them down. And this can be acknowledged only when actually investigating the subtle ways in which technologies help to shape political engagement, action, and community. Instead of applying a pre-given framework to a technology, we need to let technologies be a challenge to these frameworks.

This is not to say that philosophical notions like Stiegler’s concept of the “pharmakon” could not play a significant role in political philosophy of technology—to the contrary. But in order to be meaningful, such concepts need to be thought through, redefined, and sharpened in relation to actual technological developments. Without a micro-level analysis, the macro-level runs the risk of getting detached from what it aims to clarify.

## Marxism and Beyond

This need for an approach ‘from within’ also has implications for the role of (neo-)Marxism in political philosophy of technology. Ever since its first beginnings, Marxism has played a central role in philosophy of technology. There are even good reasons to see Karl Marx himself as one of the first philosophers of technology, laying bare how technological means of production have profound social impacts. It is especially Andrew Feenberg who has taken up this Marxist legacy and elaborated it into a sophisticated political theory of technology that makes it possible to analyze phenomena of technological instrumentalization, critical appropriation, and the democratization of design, in close dialogue with the empirical field of Science and Technology Studies.

All of these Marxist and neo-Marxist approaches have a dialectical character. They think in terms of struggle: oppression and resistance, enslavement and liberation, distraction and self-consciousness. Stiegler’s approach to human-technology relations, which plays a central role in Lemmens’ analysis, gives this dialectics a fascinating new turn. For him, the struggle between humanity and technology is constitutive for the human being. Our “originary technicity” makes technology part of the human condition, and for that reason it is a “pharmakon”: poison and medication at the same time. Curing our innate defect—the fact that human beings are born as deficient beings who have to augment themselves with technologies—implies that we have to poison ourselves in order to live.

This is a very sophisticated dialectical model, which is not simply a dialectics of oppression and liberation, but an unavoidable struggle to live with technology. For that reason, it is a bit disappointing that Lemmens concludes his analysis with the thesis that we need to appropriate technologies as “weapons (…) for (re-)conquering our collective autonomy in the face of an increasingly totalitarian capitalism bent on controlling subjects (…).” In such a diagnosis, information technologies are mere vehicles of capitalism, and dialectics is a mere fight for autonomy. This seems to ignore the hermeneutical orientation in the dialectic frameworks of Feenberg and Stiegler, in which technologies are constitutive for human beings. A call to take up arms against the technologies of capitalism reinstates a separation of human beings and technologies that goes against the most central insights in philosophy of technology and Science and Technology Studies of the past decades.

## Realism

Political philosophy of technology, therefore, is in need of *realism*. A realism in the phenomenological sense of going back “to the things themselves”. Technologies are not opposed to politics; they are its very media. The political significance of technologies cannot be reduced to its origins in specific systems of production or social organization. Technologies mediate politics: they help to shape political engagement, interactions, frameworks, power relations, self-consciousness and social awareness. And for this reason, doing political theory of technology requires close analyses of the technological things themselves, and a realistic recognition of the technological condition of human existence. Not as weapons to fight with, but as the ecostructure in which we inevitably live our lives.
